# RNA-seq based SNPs in some agronomically important oleiferous lines of *Brassica rapa* and their use for genome-wide linkage mapping and specific-region fine mapping

**DOI:** 10.1186/1471-2164-14-463

**Published:** 2013-07-09

**Authors:** Kumar Paritosh, Satish K Yadava, Vibha Gupta, Priya Panjabi-Massand, Yashpal S Sodhi, Akshay K Pradhan, Deepak Pental

**Affiliations:** 1 Centre for Genetic Manipulation of Crop Plants, University of Delhi South Campus, Benito Juarez Road, New Delhi 110021, India; 2 Department of Genetics, University of Delhi South Campus, Benito Juarez Road, New Delhi 110021, India

**Keywords:** *Brassica rapa*, RNA-seq, Next generation sequencing, Single nucleotide polymorphism (SNP), Paralog specific variation (PSV), Coding DNA Sequences (CDS), KASPar assays

## Abstract

**Background:**

*Brassica rapa* (AA) contains very diverse forms which include oleiferous types and many vegetable types. Genome sequence of *B. rapa* line Chiifu (ssp. *pekinensis*), a leafy vegetable type, was published in 2011. Using this knowledge, it is important to develop genomic resources for the oleiferous types of *B. rapa*. This will allow more involved molecular mapping, in-depth study of molecular mechanisms underlying important agronomic traits and introgression of traits from *B. rapa* to major oilseed crops - *B. juncea* (AABB) and *B. napus* (AACC). The study explores the availability of SNPs in RNA-seq generated contigs of three oleiferous lines of *B. rapa* - Candle (ssp. *oleifera*, turnip rape), YSPB-24 and Tetra (ssp. *trilocularis*, Yellow sarson) and their use in genome-wide linkage mapping and specific-region fine mapping using a RIL population between Chiifu and Tetra.

**Results:**

RNA-seq was carried out on the RNA isolated from young inflorescences containing unopened floral buds, floral axis and small leaves, using Illumina paired-end sequencing technology. Sequence assembly was carried out using the Velvet *de-novo* programme and the assembled contigs were organised against Chiifu gene models, available in the BRAD-CDS database. RNA-seq confirmed the presence of more than 17,000 single-copy gene models described in the BRAD database. The assembled contigs and the BRAD gene models were analyzed for the presence of SSRs and SNPs. While the number of SSRs was limited, more than 0.2 million SNPs were observed between Chiifu and the three oleiferous lines. Assays for SNPs were designed using KASPar technology and tested on a F_7_-RIL population derived from a Chiifu x Tetra cross. The design of the SNP assays were based on three considerations - the 50 bp flanking region of the SNPs should be strictly similar, the SNP should have a read-depth of ≥7 and no exon/intron junction should be present within the 101 bp target region. Using these criteria, a total of 640 markers (580 for genome-wide mapping and 60 for specific-region mapping) marking as many genes were tested for mapping. Out of 640 markers that were tested, 594 markers could be mapped unambiguously which included 542 markers for genome-wide mapping and 42 markers for fine mapping of the *tet-o* locus that is involved with the trait tetralocular ovary in the line Tetra.

**Conclusion:**

A large number of SNPs and PSVs are present in the transcriptome of *B. rapa* lines for genome-wide linkage mapping and specific-region fine mapping. Criteria used for SNP identification delivered markers, more than 93% of which could be successfully mapped to the F_7_–RIL population of Chiifu x Tetra cross.

## Background

The Next-Generation Sequencing (NGS) technologies are being extensively used for genome-wide genetic marker development through RNA-seq, reduced-representation sequencing, restriction-site-associated DNA sequencing (RAD-seq) and low-coverage genotyping [1]. Availability of abundant markers will facilitate association mapping, marker aided selection (MAS), and fine mapping of regions of interest for circumventing the problem of linkage drag during introgressions and for map based cloning.

NGS technologies have also contributed to completion of reference genome sequences of many important crops [2,3]. Availability of reference genomes will facilitate characterization of variability within a crop and its wild relatives by high throughput re-sequencing. In the family *Brassicaceae*, model species *Arabidopsis thaliana* was sequenced by the Sanger method using aligned overlapping BACs [4]. Using NGS technologies, a large number of ecotypes have been sequenced in a much shorter span of time [5]. Eventually 1001 ecotypes will be sequenced.

The first crop species sequenced from *Brassicaceae* is *Brassica rapa* (2n = 20, AA genome) [6]. The assembled sequence of 283.8 Mb covers more than 98% of the gene space. Sequencing was carried out using Illumina GA II technology. Sequence data was integrated with BAC-end sequences obtained through the Sanger sequencing method. Sequencing work was carried out on line Chiifu, a leafy vegetable type of *B. rapa* belonging to ssp. *pekinensis.* The genome sequence of Chiifu is available on BRAD, a genomic database created for *B. rapa* and other *Brassica* species [7,8].

Genus *Brassica* contains some of the most important vegetable and oleiferous crops of the world. The relationship of the six crop species namely, *B. rapa* (AA, 2n = 20), *B. nigra* (BB, 2n = 16), *B. oleracea* (CC, 2n = 18), *B. juncea* (AABB, 2n = 36), *B. napus* (AACC, 2n = 38), *B. carinata* (BBCC, 2n = 34) was first described by U [9] and later confirmed by others using molecular markers [10]. Crop Brassicas display a range of morphotypes, which include vegetable types where root, leaves, stems and inflorescence have been modified for human consumption, oilseed types and condiment types, all selected under domestication [11]. Three of the species namely *B. juncea*, *B. napus* and *B. carinata* are recent allopolyploids with the full chromosome compliment of the two parental genomes. The three diploids are paleohexaploids with extensive chromosomal rearrangements, gene subfunctionalization and loss [6,12,13]. Comparative genomic studies have shown that the gene blocks identified in *A. lyrata* and *A. thaliana* are represented at least three times in *B. rapa*, although every gene in a block is not necessarily represented by three paralogs [12-15].

*B. rapa* as a species shows enormous morphological variability, containing both vegetable types and oilseed types, and has extensive geographical distribution [11,16]. Different morphotypes have been classified under subspecies (ssp.). There is however, no consensus on the relationship of different types [17]. A recent classification has recognised 10 ssp. in *B. rapa*[18]. The most extensive study to date on variability within *B. rapa,* conducted on 161 accessions with AFLP markers, has shown that oleiferous types of a region are closer to the vegetable types of that region rather than to the oleiferous types of the other regions, thereby implying independent domestication of the oleiferous types in many regions [16].

In the present study we have carried out RNA-seq of three different oleiferous lines of *B. rapa* namely, YSPB-24 and Tetra (both belonging to the Yellow sarson group, ssp. *trilocularis*), Candle (turnip rape, ssp. *oleifera*) and a vegetable type line Chiifu using Illumina GA II technology to find out if sufficient numbers of SNPs are available for genome-wide mapping and for fine mapping in specific regions of the genome. YSPB-24 has a typical bilocular ovary and Tetra is an interesting variant in the Yellow sarson group as it has a tetralocular ovary. The most probable region of origin of the Yellow sarson lines is Eastern India. These lines are extensively grown in this region. Candle is an oilseed line of European origin. Therefore, the study includes two closely related oleiferous lines (YSPB-24 and Tetra), which are distant from the oleiferous line Candle. All the three lines are divergent from the leafy vegetable type line Chiifu that has been sequenced recently.

RNA-seq can provide the most informative SSRs and SNPs for gene synteny based comparative genomics [19-22] and association mapping. A number of programs have been developed for SNP identification from the NGS data [23,24]. Marking SNPs in the single-copy genes has been worked out reasonably well. However, in paleoploid species like *B. rapa* we require SNPs not only for marking allelic variation but also for marking the paralogs as has been the case for more recent allopolyploid species like wheat or *B. napus*[25-28].

We report that a sufficient number of SNPs are available in the *B. rapa* single-copy genes for genome-wide mapping and in the paralogs to mark both the allelic differences and paralog specific differences to saturate a specific region with unique marker probes. This strategy for genome-wide mapping and specific-region fine mapping has been tested using a F_7_-RIL population of a Chiifu x Tetra cross using KASPar oligo technology [29]. A total of 542 SNPs have been mapped on the 10 linkage groups of *B. rapa* and 52 SNPs have been mapped in a region of LG A4, which we have recently shown to contain the *tet-o* locus that encodes the trait - tetralocular ovary [unpublished].

## Results

### Plant material and sequencing

Four *Brassica rapa* lines – YSPB-24 and Tetralocular (Yellow sarson types, ssp. *trilocularis*), Candle (ssp. *oleifera*) and Chiifu (ssp. *pekinensis*) were used for RNA sequencing. Inflorescence, with all the unopened flower buds, along with a few small leaves was used for RNA extraction. This amalgam of organs represents most of the aerial tissues of a *Brassica* plant. Paired end cDNA libraries, used for sequencing, were prepared from the poly-A containing RNA and sequenced as 2x101 nt reads on the Illumina GAIIX sequencer.

Sequencing samples of Tetra, YSPB-24 and Candle were run in two lanes of the flow cell for sequencing whereas Chiifu sample was run in only one lane. As information on more than 98% of the gene space is available for Chiifu and has been organized as CDS in the BRAD database [8], the limited Chiifu transcriptome sequencing was carried out to check the overall quality of the assembly of the raw sequencing data obtained in this study.

### Data filtering and *de-novo* assembly of the transcriptome

**Figure 1:**
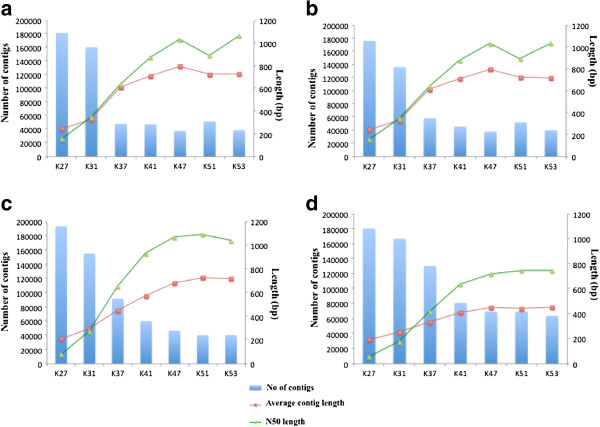
**Comparison of total contig number, average contig length and N50-length, obtained after the Velvet assembly.** Figures **A**, **B**, **C** and **D** represent the contig assembly results of *B. rapa* line Chiifu, Tetra, YSPB-24 and Candle, respectively. The bars indicate the total number of contigs assembled (primary axis). The green line represents the N50 contig length while the red line indicates the average contig length.

Paired end sequencing of transcriptome generated 84,458,126, 117,128,230, 145,049,468 and 154,228,832 sequence reads for Chiifu, Tetralocular, YSPB-24 and Candle, respectively (Table 1), the lowest being for Chiifu for which the data was obtained from single lane. After filtering low-quality and single-end reads, assembly of the cleaned reads was carried out using the Velvet *de-novo* assembly program [30] with default settings except that the minimum contig length was set at 100 bp as this length would be useful for designing oligos for SNP analysis. Reads were assembled for different K-mer values (K21 to K57) and the obtained data were analyzed for the total number of contigs, percentage of reads assembled, N50 values and the average contig length. Best assembly was found at K-mer value of 47 in case of Chiifu, Tetra and Candle and at K-mer value of 51 in case of YSPB-24 (Figure 1). The number of contigs obtained for the four *B. rapa* lines ranged from 38,220 for Tetra to 69,636 for Candle (Table 1). Approximately 61–78 percent of the reads could be assembled into contigs with N50 values ranging from 515 to 1094 bp. The maximum contig size obtained for Chiifu was 8956 bp whereas for the other three lines it was more than 10 kb. The mean depth of the line specific assemblies was found to be in the range 76.3 to 181 (Table 1).

**Table 1:** **Sequencing and assembly statistics of four different lines of *B. rapa***

	**Chiifu**	**Tetra**	**YSPB-24**	**Candle**
Total number of reads	84,458,126	117,128,230	145,049,468	154,228,832
Paired ends (after filtering low quality reads)	64,690,910	78,907,790	105,468,958	115,144,542
Number of contigs	44,972	38,220	40,422	69,636
Percentage of reads assembled	61.3	69.9	78.6	76.0
Maximum length of contigs (bp)	8,956	15,582	10,001	13,504
N50 contig length (bp)	515	1,030	1,094	719
Mean depth of the contigs	76.3	121.6	181.2	160.4

### Sequence comparison of YSPB-24, Tetra and Candle with Chiifu

We compared the Chiifu transcriptome sequence obtained in this study with the Chiifu gene models described in the BRAD database [8]. A total of 43,110 out of 44,972 contigs obtained in this study could be aligned with the CDS sequences reported in the BRAD database using the parameter of minimum 96% identity, indicating quality assembly of the raw reads in this study. However, the assembled contigs of Chiifu and the three oleiferous type lines were predominantly partial sequences (ESTs). Therefore, in the rest of the study the contigs obtained from the three oleiferous lines – YSBP-24, Tetra and Candle were compared with the more detailed full-length CDS (predicted gene models) available for the line Chiifu in the BRAD database.

Assembled sequences of each of the three oleiferous lines showing ≥ 80% sequence identity in ≥ 100 bp sequence stretch with any of the predicted gene models of Chiifu were considered to be homologous sequences. All the contigs showing identity with a Chiifu gene model as per the criteria described above were considered to be a part of the gene model and collectively referred to as a homolog. Around 90% contigs of Tetra, 88.2% contigs of YSPB-24 and 89.2% contigs of Candle matched with one or the other gene model of Chiifu listed in the BRAD database. The BRAD database lists 17,562 single-copy gene models for Chiifu in the syntenic paralog data (http://brassicadb.org/brad/searchSynteny.php). We could identify 13,808, 9,081 and 8,143 homologs in the lines Tetra, YSPB-24 and Candle, respectively (Table 2). Homologs have been grouped-‘Chiifu vs YSPB-24’, ‘Chiifu vs Tetra’ and ‘Chiifu vs. Candle’ and these have been described in the Additional file 1. Hitherto, gene models and their RNA-seq based homologs will be referred to as genes.

**Table 2:** **Homology based grouping of contigs of three oleiferous *B. rapa *lines with Chiifu**

	**Single-copy genes**	**Two paralogs**	**Three paralogs**
Chiifu*	17,562	13,506	6,645
Tetra	13,808	10,260	4,647
YSPB-24	9,081	9,400	4,515
Candle	9,343	9,604	4,692

*From BRAD database. Genes with tandem repeats were counted as one.

### SSR identification

**Figure 2:**
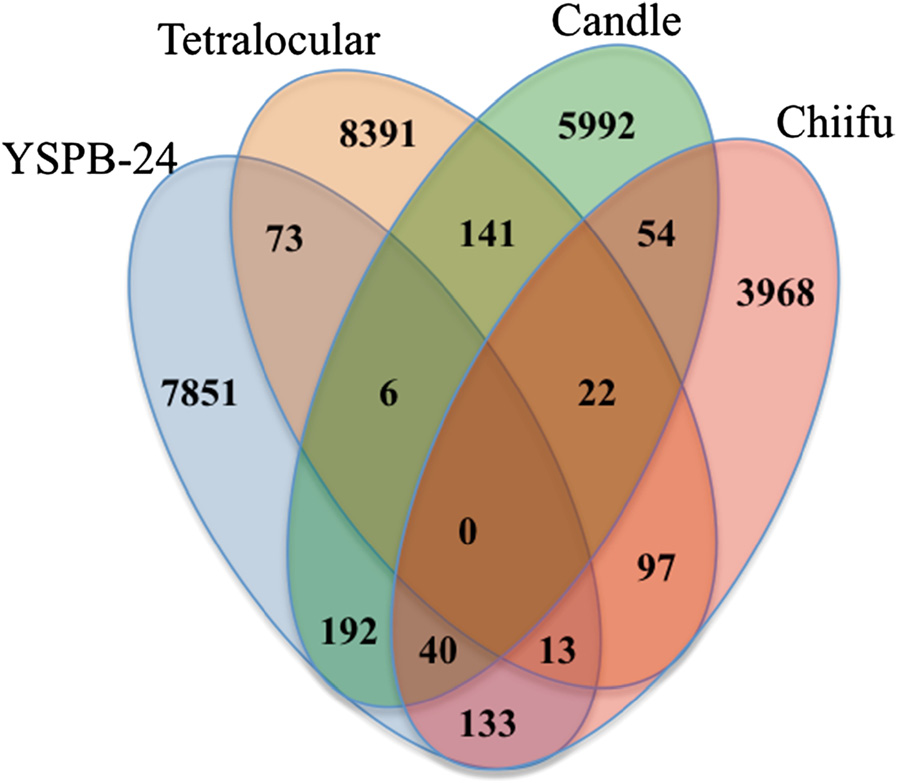
**Polymorphic SSRs between different *Brassica rapa *lines.** Venn diagram showing the number of polymorphic SSR markers available between four different lines of *B. rapa*.

The MISA program [31] was used to identify SSRs in the Chiifu gene models available in the BRAD database and in the homolog-contigs of the three oleiferous lines. The minimum cut off for the identification of mono-, di-, tri-, tetra-, penta- and hexanucleotide SSRs was set at 10, 6, 5, 5, 5 and 5 repeats, respectively. SSRs were found in 4327, 8308, 6160 and 5296 genes of Chiifu and in their homologs in Tetra, YSPB-24 and Candle, respectively. Mono-, bi- and tri-nucleotide motifs were found to be the most abundant in the three different lines (Table 3). We have recorded more mono- and di- SSRs in the contigs of Candle, YSPB-24 and Tetra as compared to the SSRs present in the gene models described in the BRAD database for Chiifu. This could be due to the presence of UTR sequences in the RNA-seq data while the CDS in BRAD database contains only those sequences that are translated into a protein. When different lines were compared *in silico* for the identification of polymorphic SSRs using the stringent criteria of complete identity in 50 bp flanks on either side of the repeat motif, most of the SSRs were found to be monomorphic. The maximum number of polymorphic SSRs (238) were found between Candle and YSPB-24 and the minimum number of polymorphic SSRs (92) were identified between Tetra and YSPB-24. The number of polymorphic SSRs identified between all the four lines in various combinations is shown in Figure 2.

**Table 3:** **Number of repeat motifs identified in the gene models of Chiifu and transcriptome sequences of the three lines of *B. rapa***

	Chiifu*	**Tetra**	**YSPB-24**	**Candle**
Mono-	150	2,087	2,028	1,282
Bi-	172	2,097	1,997	1,885
Tri-	3,976	2,634	2,455	2,859
Tetra-	3	32	36	32
Penta-	2	12	16	9
Hexa-	29	17	13	18
Complex	192	472	420	361

* Sequences analysed were taken from the BRAD- Brapa_CDS dataset.

### Identification of SNPs between different lines of *B. rapa*

Two different programs Maq [32] and MUMmer [33] were used separately to identify the single nucleotide variations between different lines of *B. rapa*. For the Maq based SNP identification between Chiifu and the other three lines, the Chiifu CDS sequence was taken as the reference and the short reads of each of the three oleiferous lines (Additional file 1) were assembled independently on the reference. The SNPs thus obtained were filtered using SNPfilter script and SNPs with less than read depth of 7 and a quality score of less than 40 were discarded. This yielded 240,424, 277,237 and 346,189 SNPs for ‘Chiifu vs. Tetra’, ‘Chiifu vs. YSPB-24’ and ‘Chiifu vs. Candle’, respectively (Table 4a). The identified SNPs were sorted based on whether these were present in single-copy genes, or in genes with two or three paralogs.

**Table 4:** **Number of SNPs identified between Chiifu and three lines of *B. rapa *using Maq and MUMmer tools**

	**Total SNPs (filtered)**	**Single-copy genes**	**Two copy genes**	**Three copy genes**
**(a) Maq software output**				
Chiifu vs. Tetra	240,424	99,845	98,740	41,839
Chiifu vs. YSPB-24	277,237	109,985	108,679	46,276
Chiifu vs. Candle	346,189	137,934	136,632	56,873
Tetra vs. YSPB-24	28,768	10,765	11,823	6,180
Tetra vs. Candle	201,827	88,426	81,127	32,274
YSPB-24 vs. candle	258,201	104,678	107,860	45,663
**(b) MuMmer tool output**				
Chiifu vs. Tetra	249,671	120,504	90,431	31,840
Chiifu vs. YSPB-24	231,259	113,408	82,699	29,376
Chiifu vs. Candle	266,349	105,498	83,983	30,485
Tetra vs. YSPB-24	20,310	10,569	7,457	2,284
Tetra vs. Candle	182,952	106,078	59,974	16,900
YSPB-24 vs. candle	176,149	102,255	58,096	15,798

When homologs of different *B. rapa* lines (Additional file 1) were analysed using the MUMmer tool, 249,671, 231,259 and 266,349 SNPs were identified between ‘Chiifu and Tetra’, ‘Chiifu and YSPB-24’ and ‘Chiifu and Candle’, respectively (Table 4b). These were further categorized on the basis of their being present in single-copy genes, or in genes with two or three paralogs.

For the identification of SNPs between lines other than Chiifu, homologs were compared in pair wise combinations of ‘Tetra vs. YSPB-24’, ‘Tetra vs. Candle’ and ‘YSPB-24 vs. Candle’ using both Maq (Table 4a) and MUMmer programs (Table 4b). Very high SNP frequencies were found for Candle vs. the two Yellow sarson lines. The least amount of polymorphism (20,310 SNPs with the MUMmer program) was recorded between Tetra and YSPB-24, the two closely related lines.

**Figure 3:**
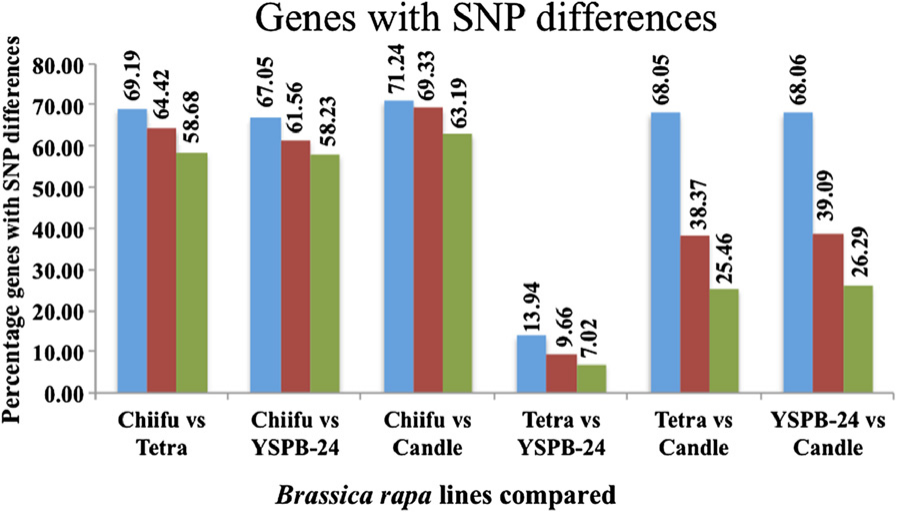
**Percentage of genes that were marked with SNPs.** Percentage of single-copy genes (first column), genes with two paralogs (second column) and genes with three paralogs (third column), which could be tagged with at least one SNP marker in pair-wise comparison of different lines of *B. rapa*. The least number of SNPs were found in Tetra vs. YSPB-24 comparison.

For the purpose of genome-wide mapping studies, one SNP is enough for marking a gene. We, therefore, carried out pair-wise analysis of SNPs in single-copy genes and in genes with two or three paralogs of all the four lines (Table 4). Except for ‘YSPB-24 vs. Tetra’ all others comparisons showed that 60-70% of the single-copy genes contained at least one SNP (Figure 3). For the two closely related lines YSPB-24 and Tetra, only 2,557 homologs could be identified with at least a single SNP - marking 14% of the single-copy genes, 9.6% of the two-gene paralogs and 7.2% of the three-gene paralogs. This number is sufficient for genome-wide linkage analysis but may turn out to be insufficient for fine mapping in a specific region.

In general, the number of SNPs recoded when the three oleiferous lines were compared with each other was lesser than the number recorded when the assembled contigs were compared with the full-length Chiifu CDS sequences available in the BRAD database (Table 4). As the contigs generated through Velvet assembly in the study were partial sequences, increasing the coverage could provide more SNPs for fine mapping.

### Marker development from the identified SNPs

A survey of the BRAD database showed that 42% of the gene models have been reported as single-copy genes and these are well distributed throughout the *B. rapa* genome (Additional file 1). Developing markers from the single-copy genes for genome-wide linkage analysis was therefore considered to be the ideal strategy.

We used Chiifu vs. Tetra polymorphism data for SNP-marker development as these could be tested on a F_7_-RIL population of Chiifu x Tetra developed in our laboratory. SNPs were identified using the following criteria

•SNPs should be surrounded with a conserved flanking region of ≥50 bp on both sides. This length of sequences would allow flexibility in positioning the oligos for amplification.

•The region of 101 bp, containing the variable base at the middle, should not have any exon-intron junctions.

•Read-depth of each of the identified SNPs should be ≥7.

A total of 6,451 single-copy genes that showed ≥ 90% sequence identity between Chiifu and Tetra were compared for SNPs. Applying the first criteria of 50 bp conserved sequences around the SNP, the number of useful contigs was reduced to 4,990. The number got reduced to 2,836 when sequences containing the intron/exon junctions were removed. Further application of the read-depth criteria reduced the number of contigs to 2,113, which could be used for marker development. To test our selection criteria for SNP marking, a set of 580 sequences, all representing single-copy genes that are well distributed throughout the genome were selected for developing SNP assays.

For specific-region mapping, the region around the *tet-o* locus was targeted. This locus has been earlier mapped (unpublished) on LG4 (A4) in a region that contains the S and I blocks of this linkage group. Sixty genes from this area were selected for developing SNP markers of which 42 were single-copy genes and 18 had two or three paralogs present in the genome. For such multi-copy genes, the paralogs were aligned and both the allele-specific SNP and paralog-specific variations (PSVs) were marked. Allele specific SNPs were selected wherein the 50 bp on either side of the SNP had some paralog specific variations (PSVs), so that locus specific primers could be designed for marking the specific paralog of interest.

Oligos for SNP genotyping were synthesized by KASPar technology [http://www.kbioscience.co.uk] using FRET quencher oligos competitor allele specific arrays. A total of 640 SNP based markers were developed between Chiifu and Tetra lines. The sequence of the marker oligos are provided in Additional file 2.

### Linkage mapping in Chiifu x Tetra F_7_ RIL population

**Figure 4:**
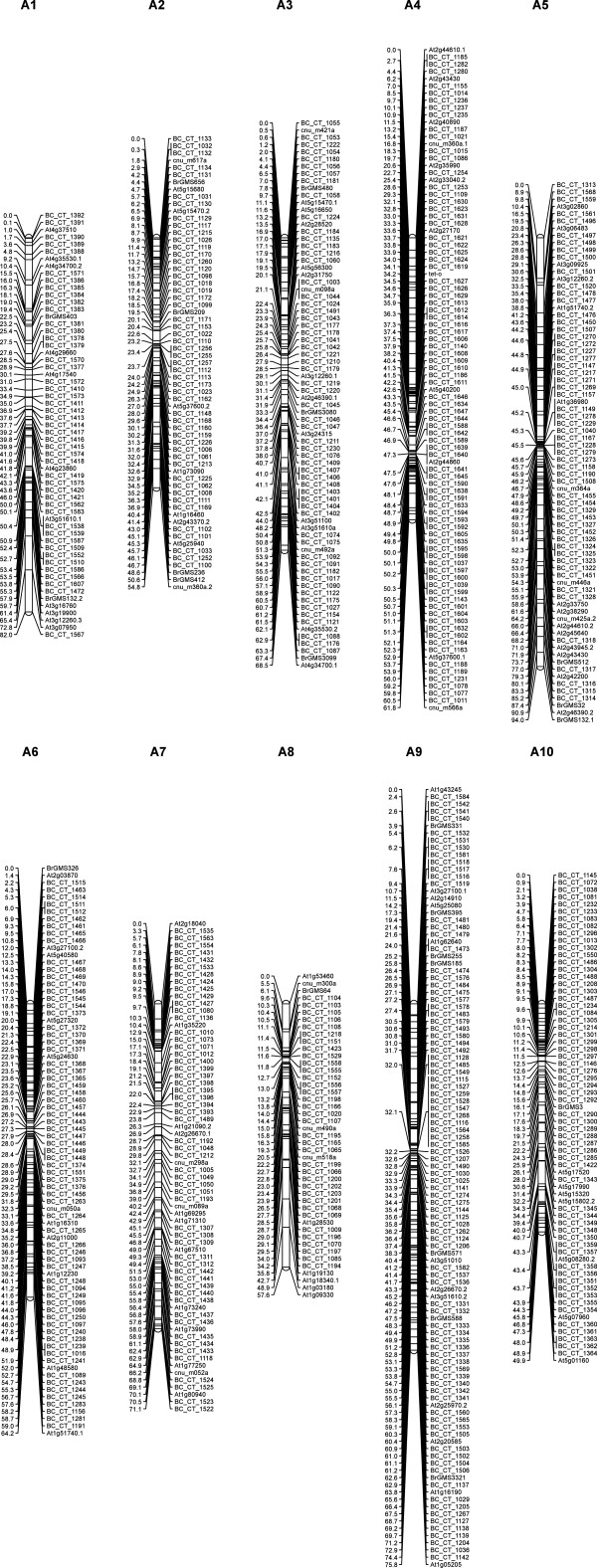
**Linkage map for *B. rapa *developed from F**_**7**_**-RILs with SNP, IP and SSR markers.** A total of 594 SNP, 39 SSR and 99 IP markers were used for the development of the linkage map. Linkage groups are named A1-A10. Markers are shown on the right of the linkage group bar and marker positions (cM) are on the left. SNP markers are with the prefix BC_CT_.

SNP marker assays were tested on a Chiifu x Tetra F_7_-RIL population of 93 individuals. Out of 640 SNPs selected from well-dispersed genes, successful assays could be obtained with 613 SNPs using KASPar genotyping technology. Twelve of the markers showed significant segregation distortion and four were found to be monomorphic. A total of 733 markers including 594 SNP markers generated in this study, 99 Intron Polymorphism (IP) markers, 39 SSR markers and one morphological marker (*tet-o*) were mapped onto the 10 linkage groups corresponding to the haploid chromosome number of *B. rapa* (Table 5). The assignment of names to the linkage groups was based on the earlier mapping work [15]. The map covered a total genetic length of 679.7 centiMorgans (cM). The markers were distributed over 653 intervals and the distance among consecutive markers ranged from 0.8 cM to 1.5 cM with an average distance of 1.1 cM. The use of well-dispersed SNP markers allowed excellent general coverage of the linkage groups. The number of markers varied from 45 (linkage group A8) to a maximum of 108 (linkage group A9) SNP markers. A linkage map based predominantly on the SNP markers is given in Figure 4.

**Table 5:** **Characteristics of 10 linkage groups of *B. rapa *map constructed with 594 SNP and 138 IP and SSR markers**

**LG**	**Length (cM)**	**Total number of markers**	**SNP markers**	**No. of intervals**	**Average interval size (cM)**
A1	82.0	59	46	54	1.5
A2	54.8	63	50	59	0.9
A3	68.5	76	58	69	1.0
A4^#^	51.8	92	80	77	0.7
A5	94.0	75	55	64	1.5
A6	64.2	79	67	77	0.8
A7	71.1	68	54	66	1.1
A8	57.6	45	35	41	1.4
A9	75.8	108	89	87	0.9
A10	49.9	68	60	60	0.8
Total	669.7	733	594	654	1.1

# The total numbers of markers includes one morphological marker *tet-o*.

**Figure 5:**
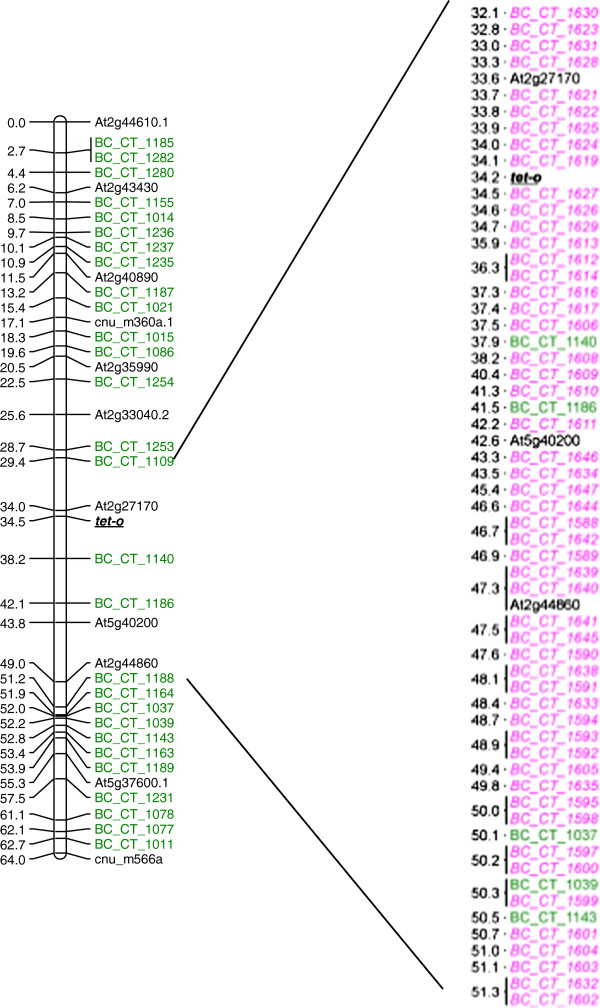
**Fine map of the *tet-o *locus.** SNP markers mapped in the region of *tet-o* locus of *B. rapa* CTF_7_ population. A total of 52 SNP markers were mapped in the region. The map on the left was developed with the SNP markers for genome-wide mapping, the *tet-o* region specific markers and their map distances in cM are shown in the map on the right side.

We further tested the use of SNP markers for fine mapping of a specific-region containing the locus *tet-o*. Of the 60 SNP markers developed for fine mapping in the region containing the *tet-o* locus, 52 could be successfully mapped with a mean marker interval of 0.5 cM. All the 18 markers designed for the genes with paralogs, marking both the SNP and PSV information for allele and paralog discrimination could be mapped without any ambiguity. The detailed map of the region containing the *tet-o* locus is shown in Figure 5.

We have earlier developed a linkage map of three different mapping populations in *B. rapa* – Chiifu x Tetra RIL-F_6_, Chiifu x Tetra F_2_ and Chiifu x YSPB-24 F_2_ using IP (genic) and SSR makers (mostly non-genic). These maps have now been integrated with the SNP map. A comparison of the features of the four parental maps, i.e. CTF_2_, CTF_6_, CYF_2_ and CTF_7_ is given in Additional file 3. IP and SSR markers genotyped on CTF_7_ in this study have been mapped previously in all these three mapping populations (unpublished) and these were used as anchor markers for developing the integrated map.

The integrated map has a total of 1,036 markers (211 IP, 230 SSR and 594 SNP) and a morphological marker ‘*tet-o*’. The features of the integrated map have been described in Additional file 4. The total genetic length spanned by the 10 linkage groups of the integrated map was 831.0 cM. The new integrated map is shown in Additional file 5.

## Discussion

Although NGS technologies can be used in a variety of ways for mapping, we have opted in this study to use them for testing whether RNA-seq could provide adequate genic SSRs and SNPs for both genome-wide linkage analysis and for fine mapping of a specific region. Our results show that the number of polymorphic genic SSRs is rather limited, but abundant SNPs are available between the distantly related (Chiifu and Tetra, Chiifu and YSPB-24 and Chiifu and Candle) lines. The extent of polymorphism is low between the two closely related lines, YSPB-24 and Tetra. In general, the numbers of SNPs available are adequate both for genome-wide mapping and specific-region fine mapping.

A number of techniques are available for marking SNPs [34]. These have been broadly classified as allele specific hybridization, allele-specific single-base extension and allele specific enzymatic cleavage. Different methods have been developed for detection of allele specific products and a number of technology platforms have been developed for allele based sequence determination. All the technologies have been used in one or the other study of SNP based mapping in plants. We chose KASPar technology as it seems to be the most appropriate technology for the most frequently encountered mapping situations in crop genetics – (a) relatively small populations and a reasonable number of markers for genome-wide linkage mapping and (b) large populations and a small number of markers for specific-region fine mapping. Markers for 640 SNPs were developed and 594 could be successfully mapped. The technology also allows development of SNP markers that can differentiate paralogs and allelic differences in one reaction.

Considerable difficulty has been encountered in marking allelic SNPs in allotetraploid species like *Triticum aestivum* (wheat) [28,35,36], *Gossypium hirsutum* (cotton) [37] and *B. napus* (rapeseed) [27] due to the presence of homeologous chromosomes [26]. *B. rapa*, in contrast to the three species mentioned above, is an ancient paleoploid with three genomes that have gone through extensive gene fractionation and chromosomal rearrangements. At the genomic level there has been extensive gene loss, leading to many genes being present as single-copy genes besides some having two or three paralogs [6].

The data available for Chiifu in the BRAD CDS database and NGS based RNA-seq carried out in this study show that a very large proportion of genes present in *B. rapa* (42% in Chiifu) exist as single-copy genes and the nucleotide polymorphism that exists between the single-copy genes of various lines is sufficient for genome-wide as well as specific-region fine mapping. Sufficient nucleotide level polymorphism also exists between the paralogs to mark these through PSVs. In our study, > 92% of identified SNPs could be converted to successful assays using the KASPar technology. Our success with KASPar markers is higher than what has been reported in wheat using this technology [36]. The possible reasons for this could be – (a) a reference genome is available in *B. rapa*, (b) the frequency of single-copy genes in the genome is very high and (c) more stringent criteria were used in this study for developing SNP detection assays.

Use of molecular markers for genetic mapping in *B. rapa* began with an extensive use of RFLP markers [38]. Use of AFLP and SRAP markers provided more extensive marker densities [39,40]. These markers, though abundant, are anonymous and do not provide any information on genomic synteny and therefore, are difficult to use for fine-mapping. Markers obtained from the gene space of a species, are most informative. EST-cDNA probes [14,41], genic SSRs [42,43], intron polymorphism (IP) markers [15,43] and InDels (Sequence Tagged Sites, STS) [44], have all been successfully used for comparative mapping and studying genomic synteny amongst the *Brassica* species belonging to the U’s triangle [9].

The large number of SNPs available in *B. rapa* will allow more involved genome-wide linkage mapping and also association studies. A core set of 168 *B. rapa* lines has been identified and used in a genome wide association study using predominately AFLP markers [45]. It should be possible to use SNPs in future genome-wide association studies. However, we expect that major emphasis in *Brassica* species will be on mapping specific traits like disease [46-49] and pest resistance and QTL for yield [50-52] through the use of populations derived from biparental crosses. This is evident from extensive work that has been carried out on mapping of such traits using biparental crosses. Precise introgressions while avoiding linkage drag, particularly when unadapted germplasm is used, is going to be the key to improvement of a large number of vegetable and oleiferous crops available within the *Brassica* species belonging to the U’s triangle. The ready availability of SNPs for both background selection (general markers) and precise introgression (specific-area markers) will help in introduction from unadapted to adapted germplasm and from the diploid species to allotetraploid species. Specific-area marking and mapping of genes, as has been shown in this study for the region containing the *tet-o* locus, will also be useful for map based cloning.

## Methods

### Plant material, RNA extraction and library preparation

The four *Brassica rapa* lines - YSPB-24, Tetralocular (Yellow sarson types, ssp. *trilocularis,* seeds procured from Indian Agriculture Research Institute, India) Candle (ssp. *oleifera,* seeds procured from Gerhard Rakow, Agri-Food, Canada), Chiifu (ssp. *pekinensis,* seeds procured from Lim Yong Pyo, Chungnam National University, South Korea*)* used for transcriptome analysis were grown in the field during the mustard growing season (October – March). Tissues for RNA isolation were taken from the field-grown plants at the time of flowering.

Inflorescence with unopened flower buds along with a few small leaves, was used for RNA extraction. Harvested tissues were immediately frozen in liquid nitrogen. Total RNA was isolated using Total RNA Spectrum Kit (Sigma), following the manufacturer’s instructions. Contaminating DNA was removed by DNase treatment (DNaseA Kit, Ambion). RNA was further purified by treatment (thrice) with acidic phenol: chloroform (1:1). RNA was quantified using Nanodrop ND1000 spectrophotometer (Nanodrop Technologies). Integrity of the obtained RNA samples was checked on Agilent 2100 Bio analyzer. RNA samples with RIN value ≥ 7 were used for further experiments.

Paired end cDNA libraries, used for sequencing, were prepared from 20 μg of total RNA using predominantly the reagents available in the mRNA-seq Sample Preparation Kit (Illumina). mRNA was isolated from the total RNA with magnetic oligo (dT) beads. Purified mRNA was fragmented by treatment with divalent cations for 5 min (solution is provided in the kit). The obtained mRNA fragments were transcribed into first strand cDNA using Superscript II reverse transcriptase (Invitrogen), followed by second-strand cDNA synthesis using DNA polymerase and RNaseH. Double stranded cDNA molecules were purified by a QIAquick PCR purification kit (Qiagen). End repair of the double-stranded cDNA was carried out using T4 DNA polymerase, the Klenow DNA polymerase and T4 polynucleotide kinase. DNA was purified again using a QIAquick PCR purification kit. This was followed by a single ‘A’ base addition at the 3’ end of the double stranded cDNA molecules using Klenow 3’ to 5’ exo-polymerase followed by purification of modified cDNA molecules using a MinElute PCR purification kit (Qiagen). Sequencing adaptors were ligated to the ‘A’ tailed fragments using T4 ligase. Adaptor ligated cDNA fragments were separated on 2% agarose gel and fragments ranging in size from 200–250 bp were excised from the gel and purified using a QIAquick Gel Extraction Kit. PCR was performed for 15 cycles from the purified DNA molecules using the adaptor specific primers (available in the Illumina kit) and the amplified fragments were purified using a QIAquick PCR Purification Kit. The size and quantity of the obtained enriched cDNA libraries was checked on an Agilent 2100 Bio analyzer. Libraries with single discrete band of 200–250 bp were selected for sequencing reactions. The libraries were sequenced as 2 x 101 nt paired end reads on the Genome Analyzer IIx instrument (GAIIx, Illumina).

### Data filtering and *de-novo* assembly of the transcriptome

Data obtained from the sequencer were processed for image deconvolution and quality value calculation of each base using the CASAVA package [Version 1.6, Illumina]. Quality check was done using Fastx-toolkit [53]. Reads with more than 30% bases having a Phred quality score < 20 were removed from the analysis using fastq_quality_filter (−q 20, -p 70). Thirty-one bases of the tail region, which were found to have low Phred score, were removed from the obtained sequences using Fastx-quality_trimmer (−t 31).

The assembly of the filtered reads was done using Velvet *de-novo* assembly program with the velvetg main parameters: -ins_length_sd 20 -ins_length (variable, depending on the insert length) -read_trkg yes -min_contig_lgth 100 -scaffolding yes -alignments yes. Obtained contig sequences are available on request.

### SNP marker development

For marker development SNPs were mined from the EST contigs of Tetra generated in this study and the reference gene model sequence data of Chiifu available in the BRAD database. For identifying SNPs in the single-copy genes, the reference sequence dataset of 17,572 single-copy gene sequences was compared against corresponding Tetra contigs identified as single-copy genes. Sequences showing ≥ 90% homology were compared for SNPs using the MUMmer software. After removing sequences with insertions/deletions, sequences with SNPs and 50 bp conserved flanking sequence on both sides of the SNP were excised from the contigs using custom perl scripts. For identifying allele specific SNPs in genes with two/three paralogs, the Tetra contigs were compared against the reference sequence of Chiifu by conducting a BLAST search [54]. The paralogs were separated based on the homology and paralog specific variations (PSVs). The allelic variations were marked using custom PERL scripts and SNPs with 50 bp on each side of SNP were excised as before. PSV containing sequences were selected to design paralog specific primers. As the SNPs were identified using EST sequences, BLAST search was conducted with all the excised 101 bp sequences containing SNPs against the *B. rapa* genome sequences of Chiifu available in the BRAD database (Brapla_genome_data_v2.1) to identify intron/exon junctions. Selected SNPs showing the presence of such boundaries in the flanking regions were discarded, as the oligo designing at such positions would result in assay failure.

The cut-off read depth for the identified SNP was set at ≥ 7. For this, a Velvet assembly generated .afg file was converted to .ace file and further pileup file was generated using Samtools [55]. The depth of each of the base (A, T, G and C) was calculated using custom perl scripts.

### SNP genotyping and construction of linkage map

For validation and mapping of SNPs, KBioscience KASPar assay [29] was used. The primer design and assay development was undertaken by KBiosciences [29]. The ID and features of identified putative SNPs along with 100 bp sequences are provided in the Additional file 2.

For genotyping the SNPs markers, a Recombinant Inbred Line (RIL) population of 93 individuals in the F_7_ generation derived from a cross between the leafy vegetable type line Chiifu and the oleiferous line Tetra was used. From the already developed map of *B. rapa* using F_6_ -RIL population of Chiifu and Tetra in our lab (unpublished), randomly selected 99 IP and 39 SSR markers, well dispersed on all the 10 linkage groups were also genotyped on F_7_-RILS to develop a skeleton map. PCR reactions and product separation for IP and SSR markers were performed as described earlier [15]. Linkage groups were established at a LOD > 6.0 with Join Map 4.0 [56] following the mapping criteria of Pradhan et al. [57]. The recombination fractions were transformed to map distances with the Kosambi function [58]. The graphical representation of the linkage groups was generated by Map Chart 2.2 [59].

For the construction of an integrated map, marker information from three mapping populations CTF_2_ (Chiifu x Tetra F_2_), CTF_6_ (Chiifu x Tetra F_6_) and CYF_2_ (Chiifu x YSPB-24 F_2_) [unpublished] along with the genotyped SNP markers was used. Recombination fraction data from the CTF_2_, CTF_6_ and CYF_2_ were merged with the recombination data of CTF_7_ map and heterogeneity tests were performed for pairs of markers common to the four mapping populations. A set of markers from each linkage group was identified on the basis of the order among the component maps (CTF_2_, CTF_6,_ CTF_7_ and CYF_2_) and was used to define a fixed order for the construction of an integrated map. Common marker pairs that differed significantly (p < 0.01) in the recombination frequencies were excluded from mapping. An integrated map was generated by Join Map 4.0 with the combine groups for a map integration function using the regression mapping algorithm.

## Conclusion

RNA-seq of three agronomically interesting oleiferous lines of *B. rapa* using paired end sequencing provided a large number of SNPs for both genome-wide mapping and for fine mapping in specific areas of the genome. High confidence SNPs in homologs were selected for KASPar based genetic marker development by three-step selection criteria. High frequency (~96%) of markers could be mapped successfully in the *B. rapa* genome. KASPar technology can be effectively used for marking allelic SNPs and PSVs for marking paralogs in *B. rapa* lines, and can be used for marking genes with multiple paralogs in any genome. Also, availability of large number of SNPs will allow fine mapping of regions of interest.

## Competing interests

The authors declare that they have no competing interests.

## Author’s contributions

KP carried out all the sequencing reactions and the bioinformatics work. VG helped with the development of the SNP markers. SKY, PP-M and AKP carried out the mapping work. YSS developed and maintained the mapping population. DP initiated the study and wrote the manuscript with inputs from all the authors. All authors read and approved the final manuscript.

## Supplementary Material

Additional file 1 **The homology based groupings of ‘Chiifu vs YSPB-24’, ‘Chiifu vs Tetra’ and ‘Chiifu vs Candle’ as a database.** The data contains the homology based grouping of *Arabidopsis* genes and their corresponding genes in *Brassica rapa* line Chiifu (as available in the BRAD syntenic paralog data) and contigs obtained for each of the three oleiferous lines of *Brassica rapa*. Column A, B, C, D, E, F, G and H represent the *Arabidopsis* gene, gene block, chromosomal position of the Chiifu homolog 1, gene id of the Chiifu homolog 1, chromosomal position of the Chiifu homolog 2, gene id of the Chiifu homolog 2 and chromosomal position of the Chiifu homolog 3, gene id of the Chiifu homolog 3, respectively. Column I onwards contain the contigs obtained from oleiferous *B. rapa* types. Click here for file

Additional file 2 **Sequences and descriptions of the oligos used for marking SNPs in the study.** The data contains the description of the genes for which the SNP based markers were developed in this study. Column B shows marker id, column C- *Arabidopsis* homolog id, column D- chromosomal position of the *Brassica* gene, column E- block position of the gene, column F- gene id in Chiifu, column G- homologous contig id in Tetra, column H- alignment direction, column I- position of the tagged SNP in the Chiifu gene sequence, column J- variable base in the Chiifu gene sequence, column K- corresponding SNP base in the Tetra contig sequence, column L- position of the tagged SNP in the Tetra gene sequence, column M- depth of the tagged base in the assembled Tetra contigs, columns N to Q- depth of the tagged base A, T, G and C at the SNP base position in assembled Tetra contigs and column R- sequence used for the marker development. The variable bases in the sequences are shown in a bracket. Degeneracy was put in some of the marker sequences. The *Arabidopsis* id and corresponding genes id for the Chiifu have been obtained from the BRAD database. Click here for file

Additional file 3 **Comparison of the features of the four parental maps, i.e. CTF**_**2**_**, CTF**_**6**_**, CYF**_**2**_**and CTF**_**7**_**, which were developed by a cross between Chiifu and Tetra lines of*B. rapa*.** Click here for file

Additional file 4 **Features of an integrated map of Chiifu x Tetra F**_**7**_**-RIL population developed by using SNP, IP and SSR markers.** Click here for file

Additional file 5 **An integrated map of CTF**_**7 **_**with 1036 markers, which include 594 SNP, 211 IP and 230 SSR markers.** Click here for file
