# Supplementary material for: RNA-seq based SNPs in some agronomically important oleiferous lines of Brassica rapa and their use for genome-wide linkage mapping and specific-region fine mapping
Source: BMC Genomics. 2013 Jul 9;14:463. doi: 10.1186/1471-2164-14-463 (PMC3711843; doi:10.1186/1471-2164-14-463)
Supplement: Additional file 3 — Comparison of the features of the four parental maps, i.e. CTF2, CTF6, CYF2and CTF7, which were developed by a cross between Chiifu and Tetra lines ofB. rapa. [file 1471-2164-14-463-S3.docx]

**Additional file 3 Characteristic features of the four parental maps used for the construction of an integrated map of *B. rapa***

| **Mapping Population** | **Cross and Population Size** | **Total Number of markers** | **Total genetic length (cM)** | **Number of intervals** | **Average interval size (cM)** | **Number of Co-dominant markers** | **Number of Dominant markers** | **Type and Number of markers** | | |
| --- | --- | --- | --- | --- | --- | --- | --- | --- | --- | --- |
|  |  |  |  |  |  |  |  | **SSR** | **IP** | **SNP** |
| **CTF_2_** | Chiifu x Tetra  (93 F_2_ plants) | 414^#^ | 813.9 | 368 | 2.5 | 252 | 162 | 211 | 202 | - |
| **CTF_6_** | Chiifu x Tetra  (94 F_6_ RILs) | 224^#^ | 851.8 | 209 | 4.3 | 159 | 65 | 116 | 107 | - |
| **CYF_2_** | Chiifu x YSPB-24 (93 F_2_ plants) | 217 | 869.8 | 199 | 4.4 | 142 | 75 | 111 | 106 | - |
| **CTF_7_** | Chiifu x Tetra  (94 F_7_ RILs) | 733^#^ | 679.7 | 653 | 1.1 | 733 | 1 | 39 | 99 | 594 |

# The total number of markers includes one morphological marker *tet-o*
