# Supplementary material for: RNA-seq based SNPs in some agronomically important oleiferous lines of Brassica rapa and their use for genome-wide linkage mapping and specific-region fine mapping
Source: BMC Genomics. 2013 Jul 9;14:463. doi: 10.1186/1471-2164-14-463 (PMC3711843; doi:10.1186/1471-2164-14-463)
Supplement: Additional file 4 — Features of an integrated map of Chiifu x Tetra F7-RIL population developed by using SNP, IP and SSR markers. [file 1471-2164-14-463-S4.docx]

**Additional File 4 Characteristic features of *B. rapa* Integrated map**

| **Linkage group** | **Length (cM)** | **Number of markers** | **Number of Intervals** | **Average interval size (cM)** | **IP** | **SSR** | **SNP** |
| --- | --- | --- | --- | --- | --- | --- | --- |
| **A1** | 84.4 | 93 | 87 | 1.0 | 19 | 28 | 46 |
| **A2** | 87.6 | 97 | 91 | 1.0 | 25 | 22 | 50 |
| **A3** | 93.8 | 133 | 117 | 0.8 | 34 | 41 | 58 |
| **A4** | 71.5 | 128^#^ | 110 | 0.7 | 29 | 18 | 80 |
| **A5** | 105.4 | 98 | 86 | 1.2 | 23 | 20 | 55 |
| **A6** | 75.5 | 101 | 99 | 0.8 | 19 | 15 | 67 |
| **A7** | 78.7 | 104 | 95 | 0.8 | 19 | 31 | 54 |
| **A8** | 70.0 | 59 | 54 | 1.3 | 8 | 16 | 35 |
| **A9** | 102.1 | 149 | 121 | 0.8 | 26 | 34 | 89 |
| **A10** | 62.0 | 74 | 65 | 1.0 | 9 | 5 | 60 |
| **Total/Average*** | **831.0** | **1036^#^** | **925** | **0.9*** | **211** | **230** | **594** |

# The total number of markers includes one morphological marker *tet-o*
