# Supplementary material for: RNA-seq based SNPs in some agronomically important oleiferous lines of Brassica rapa and their use for genome-wide linkage mapping and specific-region fine mapping
Source: BMC Genomics. 2013 Jul 9;14:463. doi: 10.1186/1471-2164-14-463 (PMC3711843; doi:10.1186/1471-2164-14-463)
Supplement: Additional file 5 — An integrated map of CTF7 with 1036 markers, which include 594 SNP, 211 IP and 230 SSR markers. [file 1471-2164-14-463-S5.pdf]

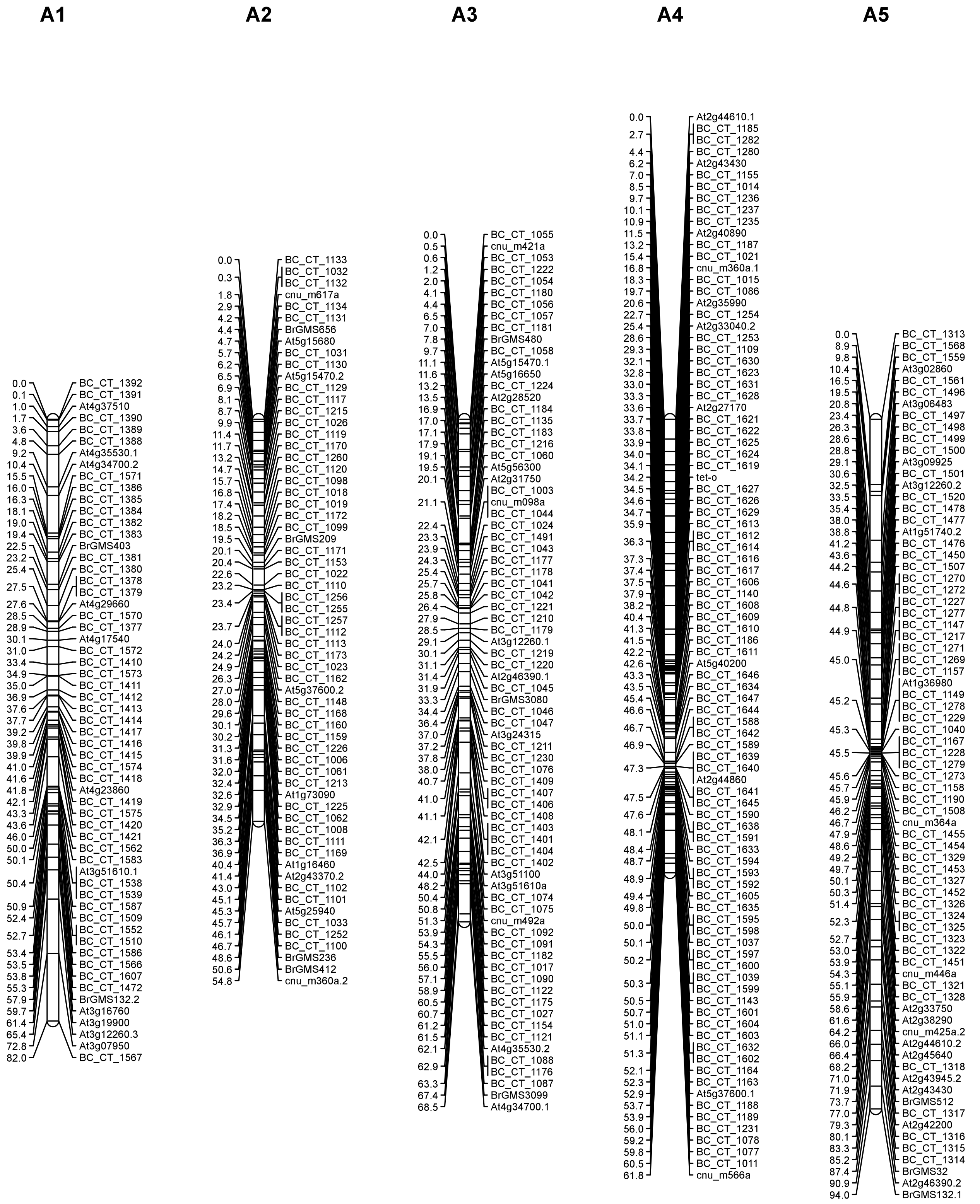

Additional File5 Linkage map for *B. rapa* developed from F7-RILs with SNP (594), IP (211) and SSR (230) markers. Linkage groups are named A1-A10. Markers are shown on the right of the linkage group bar and marker positions (cM) are on left.

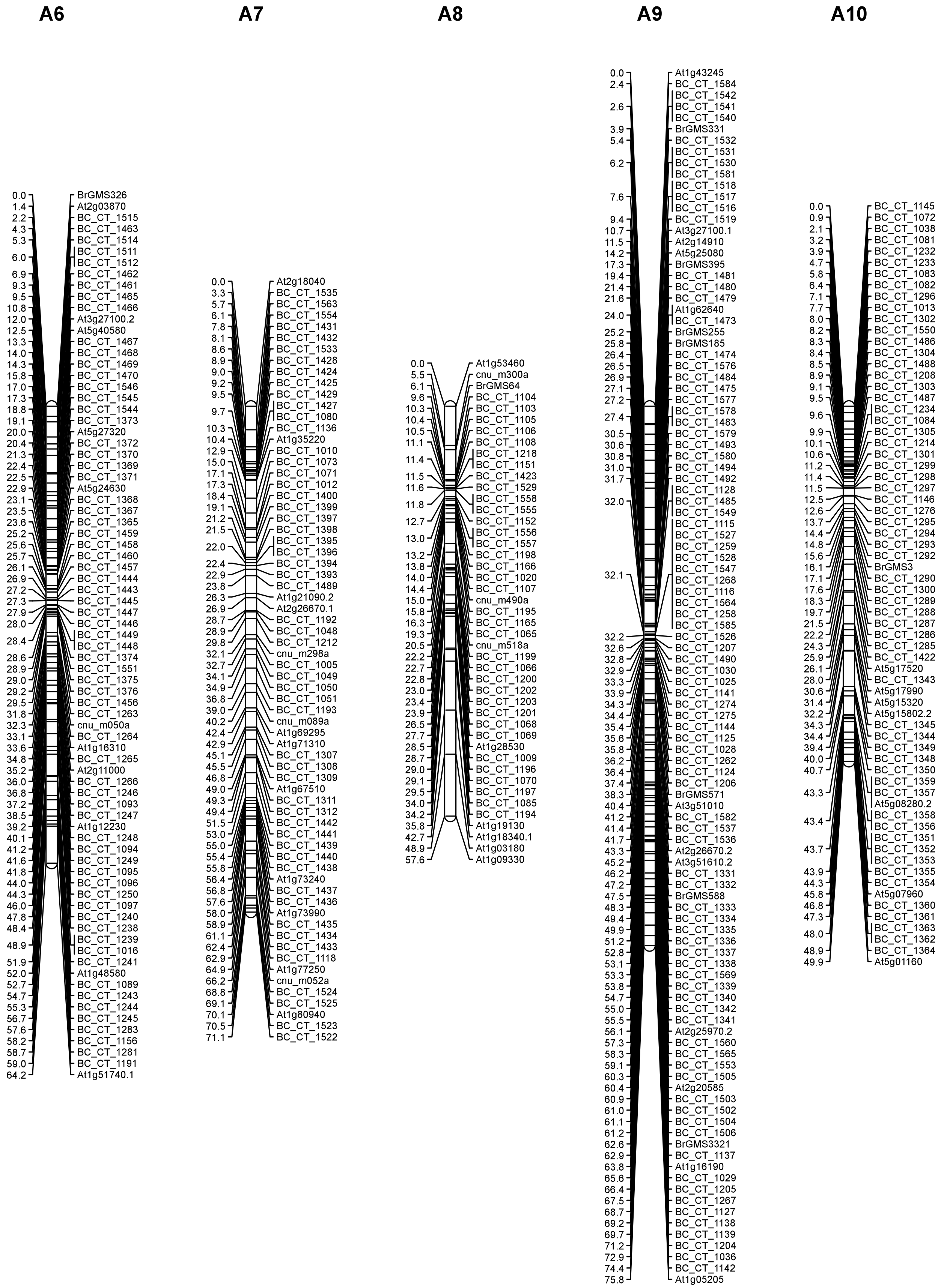
A6
0.0
1.4
2.2
4.3
5.3
6.0
6.9
9.3
9.5
10.8
12.0
12.5
13.3
14.0
14.3
15.8
17.0
17.3
18.8
19.1
20.0
20.4
21.3
22.4
22.5
22.9
23.1
23.5
23.6
25.2
25.6
25.7
26.1
26.9
27.2
27.3
27.9
28.0
28.4
28.6
28.9
29.0
29.2
29.5
31.8
32.3
33.1
33.6
34.8
35.2
36.0
36.8
37.2
38.5
39.2
40.1
41.2
41.6
41.8
44.0
44.3
46.0
47.8
48.4
48.9
51.9
52.0
52.7
54.7
55.3
56.7
57.6
58.2
58.7
59.0
64.2
BrGMS326
At2g03870
BC_CT_1515
BC_CT_1463
BC_CT_1514
BC_CT_1511
BC_CT_1512
BC_CT_1462
BC_CT_1461
BC_CT_1465
BC_CT_1466
At3g27100.2
At5g40580
BC_CT_1467
BC_CT_1468
BC_CT_1469
BC_CT_1470
BC_CT_1546
BC_CT_1545
BC_CT_1544
BC_CT_1373
At5g27320
BC_CT_1372
BC_CT_1370
BC_CT_1369
BC_CT_1371
At5g24630
BC_CT_1368
BC_CT_1367
BC_CT_1365
BC_CT_1459
BC_CT_1458
BC_CT_1460
BC_CT_1457
BC_CT_1444
BC_CT_1443
BC_CT_1445
BC_CT_1447
BC_CT_1446
BC_CT_1449
BC_CT_1448
BC_CT_1374
BC_CT_1551
BC_CT_1375
BC_CT_1376
BC_CT_1456
BC_CT_1263
cnu_m050a
BC_CT_1264
At1g16310
BC_CT_1265
At2g11000
BC_CT_1266
BC_CT_1246
BC_CT_1093
BC_CT_1247
At1g12230
BC_CT_1248
BC_CT_1094
BC_CT_1249
BC_CT_1095
BC_CT_1096
BC_CT_1250
BC_CT_1097
BC_CT_1240
BC_CT_1238
BC_CT_1239
BC_CT_1016
BC_CT_1241
At1g48580
BC_CT_1089
BC_CT_1243
BC_CT_1244
BC_CT_1245
BC_CT_1283
BC_CT_1156
BC_CT_1281
BC_CT_1191
At1g51740.1
A7
0.0
3.3
5.7
6.1
7.8
8.1
8.6
8.9
9.0
9.2
9.5
9.7
10.3
10.4
12.9
15.0
17.1
17.3
18.4
19.1
21.2
21.5
22.0
22.4
22.9
23.8
26.3
26.9
28.7
28.9
29.8
32.1
32.7
34.1
34.9
36.8
39.0
40.2
42.4
42.9
45.1
45.5
46.8
49.0
49.3
49.4
51.5
53.0
55.0
55.4
55.8
56.4
56.8
57.6
58.0
58.9
61.1
62.4
62.9
64.9
66.2
68.8
69.1
70.1
70.5
71.1
At2g18040
BC_CT_1535
BC_CT_1563
BC_CT_1554
BC_CT_1431
BC_CT_1432
BC_CT_1533
BC_CT_1428
BC_CT_1424
BC_CT_1425
BC_CT_1429
BC_CT_1427
BC_CT_1080
BC_CT_1136
At1g35220
BC_CT_1010
BC_CT_1073
BC_CT_1071
BC_CT_1012
BC_CT_1400
BC_CT_1399
BC_CT_1397
BC_CT_1398
BC_CT_1395
BC_CT_1396
BC_CT_1394
BC_CT_1393
BC_CT_1489
At1g21090.2
At2g26670.1
BC_CT_1192
BC_CT_1048
BC_CT_1212
cnu_m298a
BC_CT_1005
BC_CT_1049
BC_CT_1050
BC_CT_1051
BC_CT_1193
cnu_m089a
At1g69295
At1g71310
BC_CT_1307
BC_CT_1308
BC_CT_1309
At1g67510
BC_CT_1311
BC_CT_1312
BC_CT_1442
BC_CT_1441
BC_CT_1439
BC_CT_1440
BC_CT_1438
At1g73240
BC_CT_1437
BC_CT_1436
At1g73990
BC_CT_1435
BC_CT_1434
BC_CT_1433
BC_CT_1118
At1g77250
cnu_m052a
BC_CT_1524
BC_CT_1525
At1g80940
BC_CT_1523
BC_CT_1522
A8
0.0
5.5
6.1
9.6
10.3
10.4
10.5
11.1
11.4
11.5
11.6
11.8
12.7
13.0
13.2
13.8
14.0
14.4
15.0
15.8
16.3
19.3
20.5
22.2
22.7
22.8
23.0
23.4
23.9
26.5
27.7
28.5
28.7
29.0
29.1
29.5
34.0
34.2
35.8
42.7
48.9
57.6
At1g53460
cnu_m300a
BrGMS64
BC_CT_1104
BC_CT_1103
BC_CT_1105
BC_CT_1106
BC_CT_1108
BC_CT_1218
BC_CT_1151
BC_CT_1423
BC_CT_1529
BC_CT_1558
BC_CT_1555
BC_CT_1152
BC_CT_1556
BC_CT_1557
BC_CT_1198
BC_CT_1166
BC_CT_1020
BC_CT_1107
cnu_m490a
BC_CT_1195
BC_CT_1165
BC_CT_1065
cnu_m518a
BC_CT_1199
BC_CT_1066
BC_CT_1200
BC_CT_1202
BC_CT_1203
BC_CT_1201
BC_CT_1068
BC_CT_1069
At1g28530
BC_CT_1009
BC_CT_1196
BC_CT_1070
BC_CT_1197
BC_CT_1085
BC_CT_1194
At1g19130
At1g18340.1
At1g03180
At1g09330
A9
0.0
2.4
2.6
3.9
5.4
6.2
7.6
9.4
10.7
11.5
14.2
17.3
19.4
21.4
21.6
24.0
25.2
25.8
26.4
26.5
26.9
27.1
27.2
27.4
30.5
30.6
30.8
31.0
31.7
32.0
32.1
32.2
32.6
32.8
32.9
33.3
33.9
34.3
34.4
35.4
35.6
35.8
36.2
36.4
37.4
38.3
40.4
41.2
41.4
41.7
43.3
45.2
46.2
47.2
47.5
48.3
49.4
49.9
51.2
52.8
53.1
53.3
53.8
54.7
55.0
55.5
56.1
57.3
58.3
59.1
60.3
60.4
60.9
61.0
61.1
61.2
62.6
62.9
63.8
65.6
66.4
67.5
68.7
69.2
69.7
71.2
72.9
74.4
75.8
At1g43245
BC_CT_1584
BC_CT_1542
BC_CT_1541
BC_CT_1540
BrGMS331
BC_CT_1532
BC_CT_1531
BC_CT_1530
BC_CT_1581
BC_CT_1518
BC_CT_1517
BC_CT_1516
BC_CT_1519
At3g27100.1
At2g14910
At5g25080
BrGMS395
BC_CT_1481
BC_CT_1480
BC_CT_1479
At1g62640
BC_CT_1473
BrGMS255
BrGMS185
BC_CT_1474
BC_CT_1576
BC_CT_1484
BC_CT_1475
BC_CT_1577
BC_CT_1578
BC_CT_1483
BC_CT_1579
BC_CT_1493
BC_CT_1580
BC_CT_1494
BC_CT_1492
BC_CT_1128
BC_CT_1485
BC_CT_1549
BC_CT_1115
BC_CT_1527
BC_CT_1259
BC_CT_1528
BC_CT_1547
BC_CT_1268
BC_CT_1116
BC_CT_1564
BC_CT_1258
BC_CT_1585
BC_CT_1526
BC_CT_1207
BC_CT_1490
BC_CT_1030
BC_CT_1025
BC_CT_1141
BC_CT_1274
BC_CT_1275
BC_CT_1144
BC_CT_1125
BC_CT_1028
BC_CT_1262
BC_CT_1124
BC_CT_1206
BrGMS571
At3g51010
BC_CT_1582
BC_CT_1537
BC_CT_1536
At2g26670.2
At3g51610.2
BC_CT_1331
BC_CT_1332
BrGMS588
BC_CT_1333
BC_CT_1334
BC_CT_1335
BC_CT_1336
BC_CT_1337
BC_CT_1338
BC_CT_1569
BC_CT_1339
BC_CT_1340
BC_CT_1342
BC_CT_1341
At2g25970.2
BC_CT_1560
BC_CT_1565
BC_CT_1553
BC_CT_1505
At2g20585
BC_CT_1503
BC_CT_1502
BC_CT_1504
BC_CT_1506
BrGMS3321
BC_CT_1137
At1g16190
BC_CT_1029
BC_CT_1205
BC_CT_1267
BC_CT_1127
BC_CT_1138
BC_CT_1139
BC_CT_1204
BC_CT_1036
BC_CT_1142
At1g05205
A10
0.0
0.9
2.1
3.2
3.9
4.7
5.8
6.4
7.1
7.7
8.0
8.2
8.3
8.4
8.5
8.9
9.1
9.5
9.6
9.9
10.1
10.6
11.2
11.4
11.5
12.5
12.6
13.7
14.4
14.8
15.6
16.1
17.1
17.6
18.3
19.7
21.5
22.2
24.3
25.9
26.1
28.0
30.6
31.4
32.2
34.3
34.4
39.4
40.0
40.7
43.3
43.4
43.7
43.9
44.3
45.8
46.8
47.3
48.0
48.9
49.9
BC_CT_1145
BC_CT_1072
BC_CT_1038
BC_CT_1081
BC_CT_1232
BC_CT_1233
BC_CT_1083
BC_CT_1082
BC_CT_1296
BC_CT_1013
BC_CT_1302
BC_CT_1550
BC_CT_1486
BC_CT_1304
BC_CT_1488
BC_CT_1208
BC_CT_1303
BC_CT_1487
BC_CT_1234
BC_CT_1084
BC_CT_1305
BC_CT_1214
BC_CT_1301
BC_CT_1299
BC_CT_1298
BC_CT_1297
BC_CT_1146
BC_CT_1276
BC_CT_1295
BC_CT_1294
BC_CT_1293
BC_CT_1292
BrGMS3
BC_CT_1290
BC_CT_1300
BC_CT_1289
BC_CT_1288
BC_CT_1287
BC_CT_1286
BC_CT_1285
BC_CT_1422
At5g17520
BC_CT_1343
At5g17990
At5g15320
At5g15802.2
BC_CT_1345
BC_CT_1344
BC_CT_1349
BC_CT_1348
BC_CT_1350
BC_CT_1359
BC_CT_1357
At5g08280.2
BC_CT_1358
BC_CT_1356
BC_CT_1351
BC_CT_1352
BC_CT_1353
BC_CT_1355
BC_CT_1354
At5g07960
BC_CT_1360
BC_CT_1361
BC_CT_1363
BC_CT_1362
BC_CT_1364
At5g01160
